# Interventions Related to Takayasu Arteritis, their Clinical and Angiographic Associations, and Prognostic Relevance - A Cohort Study

**DOI:** 10.31138/mjr.030924.has

**Published:** 2025-05-15

**Authors:** Sandeep Balakrishnan, Upendra Rathore, Mishra Prabhaker, Darpan R. Thakare, Kritika Singh, Tooba Qamar, Deeksha Singh, Juhi Dixit, Manas Ranjan Behera, Neeraj Jain, Manish Ora, Dharmendra Singh Bhadauria, Sanjay Gambhir, Vikas Agarwal, Sudeep Kumar, Sudeep Durga Prasanna Misra

**Affiliations:** 1 Department of Clinical Immunology and Rheumatology, Sanjay Gandhi Postgraduate Institute of Medical Sciences (SGPGIMS), Lucknow, India;; 2 Department of Biostatistics and Health Informatics, Sanjay Gandhi Postgraduate Institute of Medical Sciences (SGPGIMS), Lucknow, India;; 3 Department of Nephrology, Sanjay Gandhi Postgraduate Institute of Medical Sciences (SGPGIMS), Lucknow, India; 4 Department of Radiodiagnosis, Sanjay Gandhi Postgraduate Institute of Medical Sciences (SGPGIMS), Lucknow, India;; 5 Department of Nuclear Medicine, Sanjay Gandhi Postgraduate Institute of Medical Sciences (SGPGIMS), Lucknow, India;; 6 Department of Cardiology, Sanjay Gandhi Postgraduate Institute of Medical Sciences (SGPGIMS), Lucknow, India

**Keywords:** Takayasu arteritis, aortic arch syndromes, interventional radiology, vascular surgical procedures, endovascular procedures

## Abstract

**Objectives:**

We analysed interventions related to TAK, their pre-treatment clinical and angiographic associations, and prognostic relevance from a large ambispective, monocentric cohort of TAK from India.

**Methods:**

Information regarding endovascular or open surgical interventions (aortoplasty, nephrectomy for refractory hypertension) was retrieved from a cohort of patients with TAK. Demographic characteristics, clinical features, and angiographic involvement were compared between patients with TAK who had undergone interventions with the rest of the cohort using multivariable-adjusted odds ratios (OR, with 95% CI). Hazard ratios were used to compare the mortality rate among TAK who had undergone interventions.

**Results:**

Among 238 patients with TAK in the cohort, 41(17.23%) had undergone 69 interventions related to TAK (64 endovascular procedures, one open surgical aortoplasty, 4 nephrectomies) across 55 sittings (a single intervention sitting in 31, two in seven, three in two, and four in one). The most common arterial territories undergoing intervention were the renal arteries (n=21), subclavian arteries (n=8), and descending thoracic aorta (n=6). Six patients with TAK required repeated interventions in the same arterial territories. Patients with TAK who underwent interventions more frequently had abdominal angina (OR 5.12, 95%CI 1.36–19.26), and less often had constitutional features (OR 0.39, 0.18–0.84) at presentation without significant differences in angiography. Survival was similar in TAK who had undergone interventions to those without (hazard ratio for mortality 0.91, 95%CI 0.23–3.55).

**Conclusion:**

About one-sixth of our cohort of TAK had undergone interventions, most often endovascular interventions. One-fourth required multiple interventions. Survival was similar in TAK with or without interventions.

## INTRODUCTION

Takayasu arteritis (TAK) is a large vessel vasculitis (LVV) more common in Asia.^1–3^ TAK more often affects young females. Patients with TAK are at an increased risk of mortality when compared with the general population. Cardiovascular disease is the major cause of mortality in patients with TAK.^4–7^ Granulomatous arteritis of the aorta and its major branches in patients with TAK eventually results in arterial wall fibrosis and stenosis or occlusion, resulting in downstream ischemia.^8^ Generally, arterial wall stenosis in TAK occurs gradually, resulting in collateral vessel formation to supply the tissues downstream. At times, such collateral vessel formation can be extensive.^9^ However, when arterial stenosis or occlusion occurs rapidly or is associated with arterial thrombosis, there results critical downstream ischemia. This can result in end-organ damage such as stroke, ischemic optic neuropathy, myocardial infarction, or mesenteric ischemia.^10–12^ The subclavian steal phenomenon might also occur in patients with TAK due to retrograde flow from the vertebral artery into the arm during periods of upper limb exercise when there is significant stenosis in the proximal part of the subclavian artery.^13^ Stenosis or occlusion of the renal arteries results in renovascular hypertension, which might be refractory to treatment with antihypertensives or result in gradual loss of renal function.^14^ Rarely, patients with TAK might develop aortic or arterial aneurysms, a pathology more often seen in patients with the counterpart LVV of Giant Cell Arteritis (GCA).^3,8^ Aneurysmal dilatation of the aortic root in patients with TAK results in aortic regurgitation. Large aneurysms in patients with TAK might also be at risk of rupture.^2^

Most patients with TAK do not require interventions due to good collateral circulation.^2^ Interventions for revascularisation of the involved arteries in patients with TAK are needed for specific indications, i.e., critical downstream ischemia involving the brain, eyes, heart, or bowel, refractory renovascular hypertension, or chronic ischemia of the limbs with resulting mismatch in supply-demand resulting in limb claudication or chronic mesenteric ischemia limiting the quality of life, subclavian steal syndrome, or repair of aortic regurgitation or aortic aneurysms at a risk of rupture.^2,15–17^ Less commonly, patients with TAK might require nephrectomy due to refractory renovascular hypertension with a shrunken, non-functional kidney.^18^ Pulmonary arteritis in TAK rarely might result in haemoptysis which requires treatment with bronchial artery embolisation.^19^ Interventions could be broadly classified as endovascular repair of the involved vessels (angioplasty, stenting, or aortoplasty) or open surgical repair.^2,15,16^ The proportion of patients with TAK undergoing interventions varies in the literature from as low as 17% to as high as >80%,^15,16,20^ largely dependent on local practices and referral bias.^10^ The choice of endovascular or open surgical intervention also depends upon the locally available expertise. A systematic review comparing open and endovascular interventions in patients with TAK reported greater restenosis rates in patients with TAK undergoing endovascular interventions, whereas open surgical interventions were associated with a greater risk of post-operative stroke.^21^

Most of the available reports focus on patients with TAK who have undergone interventions. The literature involving overall cohorts comparing patients with TAK who have undergone interventions with those not requiring interventions to identify predictors of such interventions is sparse. Therefore, we undertook a descriptive analysis of procedures related to TAK from a large single-centre cohort of patients with TAK, analysed predictors of interventions based on clinical and angiography features, and compared survival in patients with TAK who had undergone interventions with those without.

## MATERIALS AND METHODS

### Database and study variables

The information for this study was retrieved from a large single-centre ambispective cohort of patients with TAK from a vasculitis clinic operational since 2017.^7,14,22,23^ All the enrolled patients with adult-onset TAK fulfilled the 1990 American College of Rheumatology (ACR) criteria,^24^ the 2022 ACR – European Alliance of Associations for Rheumatology (EULAR) classification criteria,^25^ or the 2012 revised Chapel Hill Consensus Conference definition for TAK.^26^ Patients with paediatric-onset TAK (disease onset at 18 years of age or earlier) fulfilled the 2008 EULAR / Paediatric Rheumatology International Trials Organisation / Paediatric Rheumatology European Society classification criteria for TAK.^27^ Patients with visits after 2023 were enrolled after obtaining written informed consent, and information from visits earlier than 2023 was obtained from the vasculitis clinic files from the time of cohort entry. Data of patients with their last visit before 2023 was obtained from the vasculitis clinic files after obtaining a waiver of written informed consent from the ethics committee. Information regarding demographic details, age at diagnosis of TAK, delay to diagnosis, age at cohort entry, duration of follow-up, clinical features, and angiography (involvement of individual vessels and Hata’s angiographic subtype),^28^ disease activity at presentation [as per physician global assessment (PGA) – active/inactive, Disease Extent Index in TAK (DEI.TAK), and Indian TAK Clinical Disease Activity Score (ITAS2010)], treatment with corticosteroids and conventional, biologic, or targeted synthetic disease-modifying anti-rheumatic drugs (DMARDs), and survival at the last available visit was collected using standardised case record forms. Data regarding endovascular and open surgical interventions that the patients with TAK had undergone (type of intervention, timing of intervention before or after the diagnosis of TAK, post-procedural complications) was retrieved from the vasculitis clinic files and electronic medical records. For calculating survival, the dates of first and last recorded visits were recorded. The data for this study was censored in October 2023.

### Statistical analysis

Continuous variables were represented using means with standard deviations (SD), whereas categorical variables were represented as percentages. Comparisons were performed between pre-treatment demographic characteristics, initiation on corticosteroids, DMARDs, statins or antihypertensive drugs, and disease activity comparing patients between TAK who had undergone interventions versus those without. Continuous variables were compared using unpaired Student’s t test, whereas categorical variables were compared using Chi-squared test or Fisher’s exact test (the latter if any of the four cells had fewer than five events). Endovascular and open surgical procedures and the arterial territories where these had been performed were tabulated. Pre-treatment predictors of interventions based on clinical features, involvement of individual vessels, and angiographic subtypes were analysed using univariable logistic regression to generate odds ratios (OR) with 95% confidence intervals (95%CI). Further, multivariable-adjusted logistic regression models were constructed separately for pre-treatment clinical features and vessels involved using the variables with p-value <0.1 on univariable logistic regression.

Survival was compared between patients with TAK who had undergone interventions with the rest of the cohort using Kaplan-Meier curves. The time at risk (in years) was set from cohort entry to the last follow-up or death. The assumption of the proportionality of hazards for mortality was tested using Schoenfeld residuals (p<0.05 indicative of evidence against the proportionality of hazards). If the hazard of mortality was proportional, then the hazard ratio for mortality was calculated using Cox proportional hazards regression, otherwise, exponential parametric regression survival-time model was used to calculate the hazards of mortality for patients with TAK who had undergone interventions (compared with those who had not undergone interventions).

## RESULTS

Among 238 patients with TAK in the cohort, 41(17.23%) had undergone 69 interventions related to TAK (64 endovascular procedures, one open surgical aortoplasty, 4 nephrectomies) across 55 sittings (a single intervention sitting in 31, two in seven, three in two, and four in one) (**Table 1**). Eight of the fifty-five interventions were undertaken before a diagnosis of TAK had been made. Stenting was the most common endovascular intervention (**Table 2**). The renal arteries were the most common arterial territories to have undergone intervention, accounting for about one-third of the interventions (**Table 3**). Ten patients required multiple interventions, of whom six patients required repeated interventions in the same arterial territories. Failures or complications were noted in 9/55 (16.4%) of the procedures. Four endovascular revascularisation procedures (involving two renal arteries, one each carotid artery, vertebral artery, celiac artery, and coronary artery) failed. One patient with active disease who underwent coronary artery stenting had instent thrombosis during the hospital stay and died. The remaining four patients who developed complications underwent these interventions during periods of inactive disease. A patient who underwent stenting of both iliac arteries developed iliac artery hematoma with haemorrhagic shock which recovered with supportive management including blood transfusions. Another patient who underwent balloon angioplasty of the renal artery developed a perinephric haematoma with hemodynamic compromise requiring blood transfusions and endovascular coiling to arrest the bleeding. The haematoma subsequently was infected requiring prolonged antibiotic therapy. A patient who underwent renal artery stenting developed a pseudoaneurysm at the femoral artery puncture site with a thrombus in the common femoral vein which resolved on follow-up. Another patient who underwent nephrectomy developed a subcutaneous collection in the right iliac fossa which was secondarily infected and required antibiotic therapy.

**Table 1.** Characteristics of the cohort.

	**TAK who underwent interventions (n=41)**	**TAK who had not underwent interventions (n=197)**	p values^*^
Age at disease onset (years) Mean (SD)	23.78(9.57)	25.51(10.01)	0.311
Paediatric-onset TAK [n(%)]	15 (36.58%)	56 (28.42%)	0.299
Age at diagnosis (years) Mean (SD)	27.41(10.34)	28.6(10.84)	0.508
Age at cohort entry (years) Mean (SD)	29.17(10.75)	29.65(11.26)	0.801
Sex distribution (F:M)	31:10	141:56	0.599
Diagnostic delay (years) Mean (SD)	3.70(4.73)	3.25(4.65)	0.573
Duration of follow-up (months) Mean (SD)	64 (67.34)	38.3 (43.2)	**0.002**
Mortality (n, %)	3 (7.32%)	9 (4.57%)	0.439^b^
Treatment with corticosteroids (n, %)	33 (80.49%)	140 (71.07%)	0.218^a^
Initial dose of corticosteroids (mg/day) Mean (SD)	34.5 (14.8)	30.6 (13.8)	0.159
Initiated on DMARDs [n(%)]	30 (73.2%)	136 (69%)	0.631^a^
Received anti-hypertensives [n(%)]	31 (75.6%)	154(78.57%)	0.677
Received statins [n(%)]	8 (19.5%)	20(10.15%)	0.091
Baseline ITAS2010 [Mean (SD)]	9.71 (7.54)	10.73 (7.02)	0.403
Baseline DEI.TAK [Mean (SD)]	8.24 (6.75)	9.34 (6.00)	0.299
Active disease at baseline [n(%)]	32 (78.04%)	144(73%)	0.511

DEI.TAK: Disease Extent Index in Takayasu arteritis; DMARDs: Disease-modifying anti-rheumatic drugs; F: Female; ITAS2010: Indian Takayasu arteritis Clinical Disease Activity Score; M: Male; SD: Standard deviation; TAK: Takayasu arteritis.

* Unpaired t test for mean(SD), Chi squared^a^/Fisher’s exact^b^ for proportions.

All patients who underwent endovascular interventions received aspirin, clopidogrel, or a combination of both.

p values <0.05 are highlighted in bold.

**Table 2.** Details of interventions.

**Type of procedure (n=69)**	**Number of procedures**
**Endovascular**	
Stenting	49
Angioplasty without stenting	13
Embolisation of bronchial artery	2
**Open surgical**	
Open surgical aortoplasty	1
Nephrectomy	4

**Table 3.** Arterial territories which had undergone interventions.

**Arterial territory (n=65)**	**Number of procedures**
Carotid artery	4
Subclavian artery	8
Vertebral artery	5
Aortic arch aneurysm repair	1
Descending thoracic aorta	6
Abdominal aorta	4
Renal artery	21
Celiac artery	2
Superior mesenteric artery	4
Iliac artery	4
Coronary arteries	4
Bronchial arteries	2

An individual patient could have undergone more than one intervention or interventions in more than one arterial territory at a single sitting.

Patients with TAK who had undergone interventions had similar age at onset or cohort entry, sex distribution, and delay to diagnosis when compared with those who had not undergone interventions. Similar proportions of patients in both groups had active disease by PGA and similar DEI.TAK or ITAS2010 scores at presentation. No inter-group differences between initiation of corticosteroids or DMARDs were observed (**Table 1**). Amongst patients who had undergone interventions, DMARDs had been initiated in 30 patients. The first-line DMARDs most commonly used were methotrexate (n=12), tacrolimus (n=6), followed by mycophenolate (n=5), azathioprine (n=3), methotrexate with tacrolimus (n=1), and cyclophosphamide (n=3). The second-line DMARDs used in these patients were mycophenolate (n=2), methotrexate (n=2), azathioprine (n=2), leflunomide (n=1), and adalimumab (n=1). Among the rest of the patients who had not undergone interventions, DMARDs had been initiated in 136 patients. The first-line DMARDs most commonly used were methotrexate (n=52), tacrolimus (n=50), followed by mycophenolate (n=25), azathioprine (n=7), methotrexate with mycophenolate (n=1), and tocilizumab (n=1). The second-line DMARDs used in these patients were mycophenolate (n=17), tacrolimus (n=10), azathioprine (n=9), methotrexate (n=7), leflunomide (n=1), and tocilizumab (n=1). The proportions of patients treated with anti-hypertensives or statins were similar in both groups. All the patients who had undergone interventions had received aspirin, clopidogrel, or a combination of both (**Table 1**).

Patients with TAK who had undergone interventions were more likely to have abdominal angina (OR 5.12) and less likely to have constitutional features (OR 0.39) at presentation after multivariable-adjusted logistic regression (**Table 4**). No significant differences were observed in arterial territories involved between patients with TAK who had undergone interventions versus those without**.** Similarly, no differences were observed between the two groups for Hata’s angiographic subtypes (**Supplementary Table S1)**.

**Table 4.** Pre-treatment predictors of interventions based on clinical features.

	**Intervention vs without intervention Univariable Odds ratio (95% CI) (n = 238)**	**Intervention vs without intervention Multivariable-adjusted Odds ratio (95% CI) (n = 238)**
Constitutional features	**0.44 (0.21–0.92)**	**0.39 (0.18 – 0.84)**
Carotidynia	0.89 (0.25 - 3.22)	-
Headache	0.82 (0.35 – 1.90)	–
Syncope, dizziness, or vertigo	1.31 (0.59 – 2.89)	-
TIA/Stroke	0.95 (0.37 – 2.47)	-
Seizure	2.93 (0.82 – 10.53)	3.82 (0.98 – 14.92)
Blurring vision	1.93 (0.79 – 4.70)	-
Loss of vision	1.07 (0.22 – 5.15)	-
Pulse or BP inequality	0.81 (0.41 – 1.60)	-
Pulse loss	0.85 (0.43 – 1.68)	-
Vascular bruits	0.61 (0.30 – 1.22)	-
Claudication (upper limb)	1.17 (0.57 – 2.42)	-
Claudication (lower limb)	1.85 (0.86 – 3.97)	-
Hypertension	1.20 (0.49 – 2.91)	-
Aortic regurgitation	0.35 (0.04 – 2.78)	-
Renal failure (acute or chronic)	1.61 (0.60 – 4.31)	-
Abdominal angina	**5.33 (1.47 – 19.37)**	**5.12 (1.36 – 19.26)**
Chest pain	1.30 (0.49 – 3.42)	-
Heart failure	0.91 (0.33 – 2.54)	-

* Chi squared^a^/Fisher’s exact^b^ for proportions p values <0.05 are highlighted in bold.

**Figure 1.**
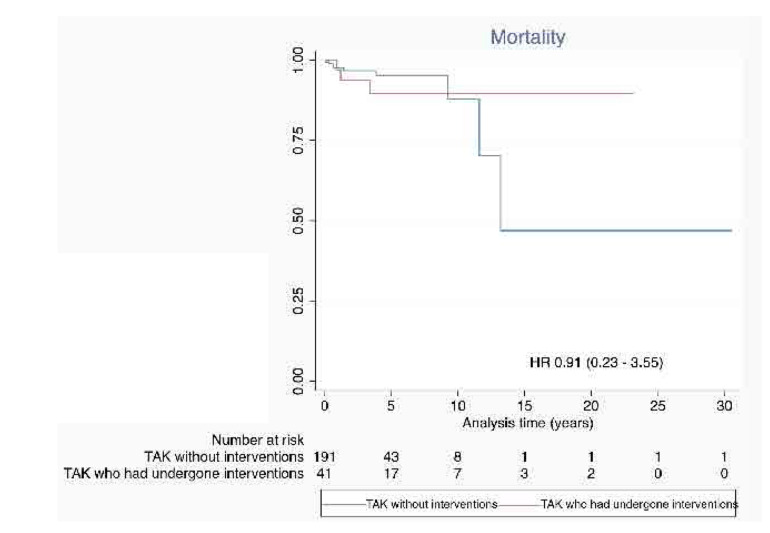
Survival in patients with Takayasu arteritis who had undergone interventions compared with those who had not undergone any intervention. HR: Hazard ratio (with 95% confidence intervals).

Similar proportions of patients died in both groups (3/41 amongst those who had undergone interventions, and 9/197 amongst those patients with TAK who had not undergone interventions) (**Table 1**). The hazards for mortality for patients with TAK who had undergone interventions or the rest of the cohort were proportional (p = 0.051 for Schoenfeld residuals). Therefore, the HR for mortality among patients with TAK with or without interventions was calculated using the Cox proportional hazards assumption. Patients with TAK who had undergone interventions had similar hazards of mortality compared with those without (HR 0.91, 95% 0.23 – 3.55) (**Figure 1**).

## DISCUSSION

In this large cohort of patients with TAK, one-sixth underwent endovascular or surgical interventions, of whom one-fourth required multiple interventions. A seventh of the procedures were undertaken before a diagnosis of TAK had been established. Failures or complications (including one fatal in-stent coronary artery thrombosis) were noted in one-sixth of procedures. Abdominal angina at presentation was associated with the requirement for interventions, whereas the presence of constitutional features was associated with a lower risk of future interventions. Patients with TAK who required interventions had a similar survival to those who did not require interventions.

The proportion of patients with TAK requiring interventions has been variously reported from different cohorts. A cohort of 97 patients with TAK from the United Kingdom (from 2001 to 2012) reported endovascular interventions in 21% and vascular surgery in 28% of patients. Restenoses were observed in 52% of endovascular interventions and 79% of open surgical interventions.^29^ Another cohort of 125 patients with TAK reported endovascular interventions in 16% (77% of which re-stenosed) and surgical bypass procedures in 29% of patients (35% of which re-stenosed).^30^ A cohort of 251 patients with TAK from the United States of America (from 1975 to 2002) reported vascular surgery in 17% of patients, one-fourth of whom required repeat procedures.^31^ A recent study from Southern India of 1149 patients with TAK (from 1996 to 2022) reported endovascular interventions in 82% of patients (re-stenoses in 49%) and open surgical interventions in 9% of patients.^20^ Compared to other published studies, this study was an outlier and had a significantly higher proportion of patients with TAK who had undergone interventions. This might also be explained by the fact that the data was based on a cohort of patients with TAK at a Cardiology centre.^20^ In comparison, our study reported interventions in 17% of patients with repeated procedures required in one-fourth of patients. The varying proportions of patients with TAK undergoing interventions from different studies reflect the variations in practice depending on the local availability of open surgical or endovascular expertise and the lack of recommendations for vascular interventions in patients with TAK.^2^

A seventh of the interventions in our cohort had been undertaken before the diagnosis of TAK had been made. Similar findings have been reported elsewhere. From an Italian cohort of patients with TAK, about 40% of procedures had been undertaken before a diagnosis of TAK had been made.^32^ This possibly reflects an underdiagnosis of TAK when patients with arterial stenosis present to Cardiologists or Interventional radiologists. One of the patients in our cohort who underwent coronary artery stenting for myocardial ischemia during active disease died following the intervention. It is imperative to correctly diagnose TAK and treat active disease before undertaking interventions as the literature also suggests those interventions undertaken during periods of active disease without peri-operative immunosuppressive therapy have a greater likelihood of restenosis or complications (including death).^2,29,31,33^ Unlike a previous study, the requirement for interventions was not associated with a greater risk of mortality in our cohort of patients with TAK.^33^

The renal arteries comprised the commonest arterial territory requiring interventions. Stenosed renal arteries might be revascularised either by stenting or balloon angioplasty. Park et al. reported similar procedural success with either stenting or angioplasty of the renal arteries. However, restenosis was lower with renal artery angioplasty (1/12) than with stenting (6/9).^34^ Kinjo et al reported similar initial success rates of revascularisation of the renal arteries with stenting, angioplasty, or bypass grafting. However, restenosis was higher with renal artery stenting (5/8) when compared with angioplasty (1/11) or surgical bypass (2/11).^35^ Peng et al. reported better initial success (primary patency 90% vs 76%) and fewer restenoses (6% vs 21%) with renal artery angioplasty than with stenting.^36^ A meta-analysis of seven studies involving patients with TAK compared 136 arterial territories that had undergone stenting with 180 that had undergone angioplasty. Overall, stenting or angioplasty had similar outcomes (OR for restenosis with stenting vs angioplasty 2.39, 95%CI 0.66–8.66) with considerable heterogeneity in the pooled estimate (I^2^ 66%). However, for the renal arteries, stenting was associated with a greater risk of restenosis without significant heterogeneity (OR 4.40, 95% CI 2.14 – 9.02, I^2^ 45%). However, fewer complications were observed with renal stenting than with angioplasty (OR 0.07, 95%CI 0.02–0.29, I^2^ 15%)21. Should endovascular intervention fail to revascularise the renal arteries, then renal autotransplantation (where the kidney is removed from its native site and reimplanted in the iliac fossa with anastomosis to the aorta) is another option that has been described for patients with TAK.^37^

Abdominal angina was identified as a significant pre-treatment predictor of the requirement for interventions in our cohort. Endovascular balloon angioplasty or stenting or open surgical repair have been reported successfully for middle aortic syndrome involving the descending thoracic aorta or the abdominal aorta, as well as for arterial lesions involving the supra-aortic arteries or the iliac arteries.^38–42^ Should stenting fail primarily or restenosis occur, drug-eluting stents might be used for revascularisation.^42^

Arteritis involving the coronary or pulmonary arteries, albeit uncommon, is well described in patients with TAK. Four patients in our cohort required interventions for coronary arteritis. Pulmonary infarcts, although rare in patients with TAK, manifest as haemoptysis which might require bronchial artery embolisation, as was required in one of our patients.^19^ The literature suggests better functional and echocardiographic outcomes (related to pulmonary hypertension) and fewer cardiac deaths with percutaneous pulmonary artery angioplasty or stenting for pulmonary artery stenosis compared with medical treatment without angioplasty.^43^ In contrast, similar survival was noted with medical treatment alone or coronary angioplasty in patients with TAK with coronary artery involvement.^44^ One study reported lesser restenosis with coronary artery bypass graft (3/12) than with percutaneous coronary angioplasty (12/19) in patients with TAK with coronary artery involvement.^45^

Few patients in our cohort underwent open surgical interventions, largely due to the greater expertise available with endovascular interventions at our centre. A systematic review with meta-analysis compared outcomes with endovascular (n=389) and open surgical interventions (n=420) in patients with TAK across 19 studies. Endovascular interventions had a higher restenosis rate when compared with open surgical interventions overall (OR 5.18, 95%CI 2.18–9.62) as well as individually for coronary arteries (OR 7.38, 95%CI 2.36–23.10), arch vessels (OR 3.97, 95%CI 1.67–9.40), and the renal arteries (OR 3.46, 95%CI 1.22–9.82) without significant heterogeneity in any of the pooled estimates. Endovascular interventions were associated with a greater risk of stroke than open surgical interventions (OR 0.33, 95%CI 0.12–0.90), however, the risk of death was similar for both (OR 0.84, 95%CI 0.38–1.85) without significant heterogeneity in the pooled estimates.^46^

Positron emission tomography (PET) is being increasingly used for the assessment of disease activity in patients with TAK.^2,47^ A point to note is that persistent 18-F fluorodeoxyglucose uptake has been observed at sites that have undergone arterial grafting and this does not necessarily indicate ongoing TAK disease activity.^48^

The strength of this study was the large number of patients with TAK. Although the data collection was done retrospectively, these patients were following up in a dedicated vasculitis clinic. Therefore, missing data was minimal. While the data regarding interventions was retrieved from the clinic files and the electronic medical records, there remains a possibility that interventions done elsewhere might have been missed. However, given that our hospital is the largest referral centre for rheumatic diseases in the region and patients are referred here with rare diseases like TAK, we think that the data regarding few if any such procedures would have been missed. The use of biologic or targeted synthetic DMARDs in our patients with TAK was sparse, however, this reflects the reality of the management of rheumatic diseases in resource-constrained settings.^49,50^

## CONCLUSION

Interventions were required in one-sixth of our cohort of patients with TAK, most often endovascular interventions. A fourth of patients required multiple interventions. The requirement for intervention did not adversely affect survival in patients with TAK.

## CONFLICT OF INTEREST

The authors declare no conflict of interest.

## FUNDING

This research did not receive any specific grant from funding agencies in the public, commercial, or not-for-profit sectors.

## STATEMENT OF ETHICS AND CONSENT

The patients included in this study were enrolled in a prospective and retrospective TAK registry. Patients enrolled prospectively were enrolled since 2023 after obtaining approval from the Institute Ethics Committee, SGPGIMS, Lucknow (document submission number 2022-152-IMP-129, date of approval 12 January 2023), and written informed consent was obtained from them. For those patients whose data was retrieved from medical records alone without direct contact with patients, waiver of informed consent was obtained from the Institute Ethics Committee, SGPGIMS, Lucknow (document submission number 2023-34-IMP-EXP-51, date of approval 03 April 2023) in line with the prevalent regulations in India given the retrospective chart review was undertaken without direct contact with patients.

## PRIOR CONFERENCE PRESENTATIONS

Presented as a poster at the 21^st^ International Vasculitis Workshop at Barcelona, Spain in April 2024.

## DATA AVAILABILITY STATEMENT

All the analyses performed for this article have been reported in the main text or the supplementary files. Anonymised data pertaining to the article shall be shared on reasonable request to the corresponding author (Durga Prasanna Misra, durgapmisra@gmail.com)

## AUTHOR CONTRIBUTIONS

Conceptualisation – DPM, UR; Data curation - DPM, SB, UR, DRT, PM, KS, TQ, DS, JD, MRB, NJ, MO, DSB, SG, VA, SK; Formal analysis - DPM, SB, UR, DRT, PM, JD, KS, TQ, DS; Funding acquisition - none; Investigation -DPM, SB, UR, DRT, PM, JD, KS, TQ, DS, MRB, NJ, MO, DSB, SG, VA, SK; Methodology – DPM, PM, SK; Project administration – DPM, UR, SK; Resources – DPM, UR, SK; Software – DPM, PM; Supervision – SG, SK; Validation - DPM, PM, SK; Visualisation - DPM, PM, SK; Roles/Writing - original draft - DPM, SB, UR, DRT, PM, JD, KS, TQ, DS; Writing - review & editing - MRB, NJ, MO, DSB, SG, VA, SK.

## Figures and Tables

**Supplementary Table S1.** Pre-treatment predictors of interventions based on angiographic involvement.

	**Intervention vs without intervention Univariable Odds ratio (95% CI)**	**Intervention vs without intervention Multivariable-adjusted Odds ratio (95% CI)**
**Vascular involvement**		
Right subclavian	0.96 (0.48 – 1.90)	-
Left subclavian	0.81 (0.40 – 1.65)	-
Right carotid	0.44 (0.21 – 0.95)	0.62 (0.26 – 1.43)
Left carotid	0.57 (0.29 – 1.14)	-
Right vertebral	**0.68 (0.08 – 5.67)**	-
Left vertebral	0.54 (0.16 – 1.89)	-
Brachiocephalic	**0.32 (0.12 – 0.87)**	0.42 (0.14 – 1.23)
Ascending aorta	0.44 (0.16 – 1.19)	-
Arch of aorta	0.76 (0.37 – 1.54)	-
Descending thoracic aorta	0.86 (0.44 – 1.69)	-
Abdominal aorta	0.61 (0.31 – 1.20)	-
Celiac trunk	0.92 (0.43 – 1.97)	-
Superior mesenteric artery	1.29 (0.62 – 2.69)	-
Inferior mesenteric artery	1.48 (0.39 – 5.62)	-
Right renal	0.95 (0.48 – 1.87)	-
Left renal	1.28 (0.65 – 2.51)	-
**Angiographic subtype**		
Hata I	0.37 (0.08 – 1.623)	-
Hata IIa	2.14 (0.53 – 8.66)	-
Hata IIb	1.30 (0.46 – 3.71)	-
Hata III	1.33 (0.36 – 5.01)	-
Hata IV	1.47 (0.51 – 4.24)	-
Hata V	0.84 (0.42 – 1.65)	-
Hata P+	0.73 (0.16 – 3.35)	-
Hata C+	2.44 (0.22 – 27.53)	-

p values <0.05 are highlighted in bold.

Iliac and femoral involvement not included in the regression models due to data sparsity.
